# Effect of Acupuncture on Time-Dependent of Muscle Endurance in Female Elbow Joint: A Randomized Controlled Trial

**DOI:** 10.1155/2022/8052256

**Published:** 2022-02-09

**Authors:** Yu Su, Shun Yao, Zi-Jie Zhou, Chou Wu, I-Lin Wang, Chien-Ying Lai

**Affiliations:** ^1^Graduate Institute, Jilin Sport University, Changchun 130022, China; ^2^Health Technology College, Jilin Sport University, Changchun 130022, China; ^3^Orthopedic Department, China Medical University Hospital, Taichung 40447, Taiwan

## Abstract

Immediate characteristics of acupuncture have been confirmed by relevant studies; however, the current study on the time effect of acupuncture in improving upper limb forearm muscle endurance is still limited. The aims of this study are to explore: (1) whether real acupuncture (RA) can improve female forearm muscle endurance compared to sham acupuncture (SA) and (2) whether the changes in forearm muscle endurance after RA are time-dependent. Thirty-six healthy female students were recruited to participate in isokinetic tests of elbow flexion/extension (Flex/Ext) from maximum flexion to maximum extension as much as possible using an isokinetic dynamometer at a speed rate of 60°/sec. Participants in the RA group were stimulated at Quchi (LI11), Shousanli (LI10), Hegu (LI4), Xiaohai (SI8), Tianjing (SJ10), and Waiguan (SJ5) acupoints for 20 min, while the SA group needling was near at these acupoints. The values of the isokinetic parameters and surface electromyography (sEMG) signals were recorded before and after acupuncture. After RA, the isokinetic parameters values (average torque, work, power, and speed), the sEMG values at four major muscles, and the joint stiffness of elbow Flex/Ext were significantly increased (*p* < 0.05). The enhancement of forearm muscle endurance lasted approximately 7–21 min (from post1 to post3/post4), indicating that the effect of RA to improve elbow Flex/Ext muscle endurance is time-dependent. Therefore, this study found that RA can immediately improve the forearm muscle endurance of healthy women compared with SA, and this effect can last approximately 7–21 min until the acupuncture efficacy decreased or disappeared.

## 1. Introduction

Acupuncture, as one of the most popular alternative therapies in Western medical practice, is often used to treat common diseases and relieve pain [1], such as acupuncture for migraine, tinnitus, chronic headache, primary insomnia, and other clinical trials have shown long-term efficacy [2]. Acupuncture has also been used in sports medicine and related disciplines to help athletes recover faster and to treat sports injuries [3] due to its small side effects, safety, and effectiveness. Therefore, acupuncture is used not only in the treatment of clinical diseases but also in sports medicine. Past studies have applied acupuncture to improve upper limb motor performance, such as acupuncture combined with neuromuscular joint facilitation (NJF) can improve shoulder ROM and activities of daily living in patients with hemiplegic shoulder pain (HSP) [4]. Therefore, acupuncture can improve the ability of upper limb movement by increasing the range of motion of upper limb joints.

Post-activation potentiation (PAP) refers to a phenomenon in which muscles have increased their functional power due to previous intense contractions [5]. Past studies have shown that professional rugby players can use the PAP effect to improve upper-limb ballistic bench press power output with sufficient recovery time [6]. Acupuncture can stimulate the skin to transmit nerve impulses, which may trigger the PAP effect and enhance the explosive force of the shoulder joint [7]. In addition, acupuncture or electrical stimulation may induce the PAP effect more easily than spontaneous muscle contraction [8]. Therefore, the application of acupuncture in athletic competitions may increase the recruitment of motor units and thereby increase the explosive power of the upper limbs.

Muscle endurance refers to the ability of muscles to maintain work during contraction, fatigue occurs after frequent muscle activity during endurance exercises, which can cause temporary hypoglycemia by disrupting the glycogen mechanism and reduce exercise performance [9]. Therefore, delaying muscle fatigue in endurance sports is essential to improve athletic performance. Past studies have shown that acupuncture can accelerate the recovery of disordered energy metabolism and choline metabolism of athletes [10]. It has been found in animal studies that acupuncture may reduce fatigue in rats during swimming by reducing inflammatory responses and inducible nitric oxide synthase expression [11]. Acupuncture at acupoints around the shoulder joint can increase the muscle endurance of the shoulder joint by increasing muscle excitability and delaying muscle fatigue [12]. Therefore, acupuncture as an alternative therapy can reduce muscle fatigue and improve muscle endurance to increase athletic performance.

The “*De-qi*” sensation experienced after acupuncture, such as soreness, numbness, heaviness, and distention, is often considered the key to achieving the therapeutic effects of acupuncture [13]. Past studies have found that acupuncture at Lingdao (HT4) acupoints can immediately weaken the ipsilateral and contralateral wrist flexor muscle strength in healthy subjects [14]. Acupuncture at Xiaohai (Si8) and Jianwaishu (Si14) acupoints can immediately enhance the electromyographic activity (root mean square) and muscle strength of bilateral upper trapezius muscles in healthy subjects [15]. Therefore, acupuncture stimulation of nerves can cause immediate changes in the total recruitment of upper limb muscle motor units, thereby affecting upper limb muscle function. Moreover, the “*De-qi*” effect will not disappear immediately after needle removal. Past studies have found that acupuncture at Quchi (Li-11) and Waiguan (TB-5) acupoints can increase the motor-evoked potential (MEP) amplitude of the first dorsal interosseus (FDI), thereby enhancing the excitability of the bilateral primary motor cortex, which can maintain within 20 min after needle removal [16]. Therefore, a period of time after needle removal may produce therapeutic effects through sustained “*De-qi*,” and the effect is time-dependent. In summary, the immediate characteristics of acupuncture have been confirmed by relevant studies; however, the current study on the time effect of acupuncture in improving upper limb forearm muscle endurance is still limited. The aims of this study are to explore: (1) whether RA can improve female forearm muscle endurance compared to SA and (2) whether the changes in forearm muscle endurance after RA are time-dependent. The hypotheses of this study are that: (1) the elbow joint muscle endurance can be improved immediately after RA compared to SA and (2) the changes in elbow joint muscle endurance after RA are time-dependent.

## 2. Methods

### 2.1. Subjects

Thirty-six healthy female students from Jilin Sport University (age: 20.64 ± 0.69 years, body mass: 58.27 ± 11.03 kg, and height: 163.17 ± 5.07 cm) were randomly divided into RA group (*n* = 18) and SA group (*n* = 18) in this trial. The selected participants maintained the following inclusion criteria: right-handed, above 18 years old, and not attending any other clinical trials within a period of 4 weeks. Individuals who have aversion to needles and diseases that affect muscle mass and strength were excluded from this study. The participants were told to perform the procedures that are described below at a scheduled time. All participants were informed about the protocol and signed freely given informed consent forms before they participated in the study.

### 2.2. Instruments and Equipment

Kinematic data on flexion/extension (Flex/Ext) of the forearm was measured by an isokinetic dynamometer (Con-Trex MJ; CMV AG, Dübendorf, Switzerland). Surface electromyography (sEMG) signals were recorded using a portable electromyography device (BTS Free EMG 300, BTS SpA, Milan, Italy) with six channels and disposable circular electrodes with a 10 mm diameter operating at a sampling rate of 1,000 Hz before and after acupuncture. Disposable stainless steel needles (0.25 mm × 40 mm, Suzhou Medical Appliance Factory, Suzhou, People's Republic of China) were inserted into specific acupuncture points during the acupuncture treatment.

### 2.3. Acupuncture

**Figure 1:**
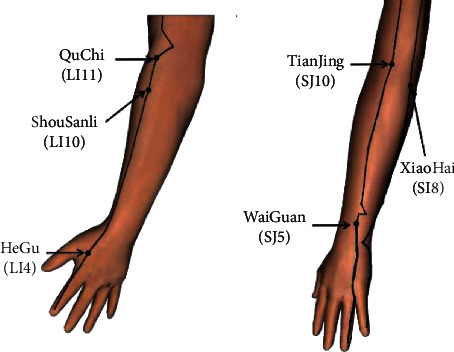
Acupuncture acupoints.

Acupuncture was performed in a sterile environment by an experienced acupuncturist with a health professional qualification approved by the Ministry of Human Resources, Social Security of the People's Republic of China, and the National Health Commission. The acupoints used by each participant are the same, including Quchi (LI11), Shousanli (LI10), Hegu (LI4), Xiaohai (SI8), Tianjing (SJ10), and Waiguan (SJ5; Figure 1), which are located near the muscles responsible for the elbow joint Flex and Ext, and acupuncture at these points can effectively activate muscle activity [17]. After cleaning the skin with alcohol, the needle was inserted perpendicularly into the acupoint for 20 min, and the needle was rotated in 90–180° at 2, 5, and 10 min after insertion to enhance the needling effects. Each point was acupunctured to achieve the “*De-qi*” sensation that indicates effective needling during acupuncture.

### 2.4. Procedure

**Figure 2:**
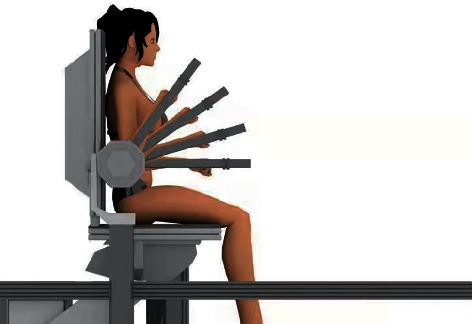
Elbow joint flexion/extension (Flex/Ext) movement.

**Figure 3:**
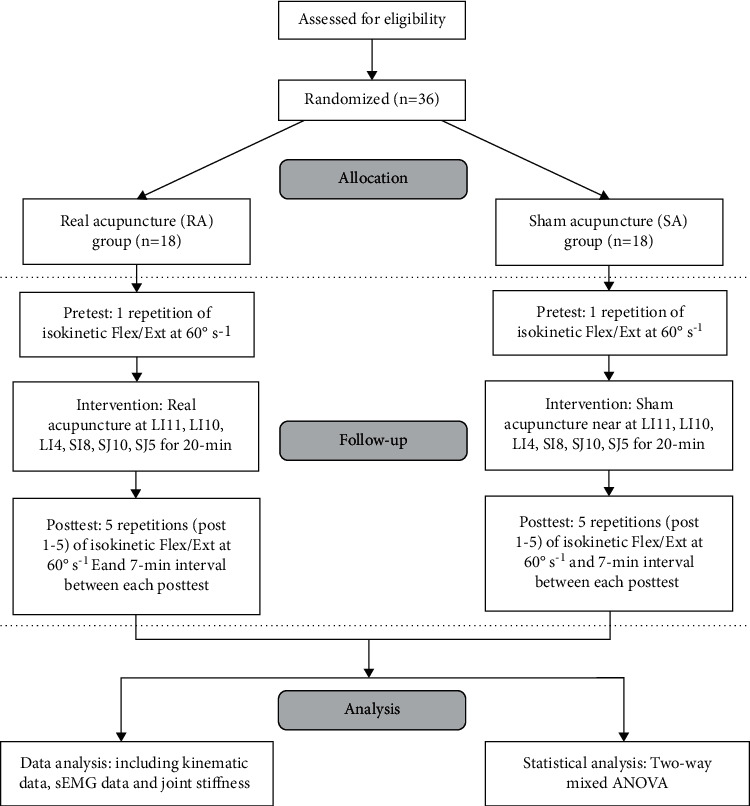
Study flowchart.

Prior to the experiment, the participants were asked to warm up and perform a set of elbow muscle stretching exercises for 10min. The surface electrodes were placed near the biceps brachii (BB), palmaris longus (PL), extensor carpi radialis longus (EC), and triceps brachii pectoralis (TB) as major elbow Flex/Ext muscles for muscle activity recording. Before the surface electrodes were placed, the skin was cleaned with 75% alcohol. The isokinetic dynamometer and the computing software were calibrated to certified weight before data collection. After a clear explanation of the test, subjects were told to seat comfortably on the isokinetic dynamometric bench with their elbows flexed 90° and the forearm in a neutral position. Besides, the trunk and forearm were stabilized using straps across the chest, waist, and wrist. The center of the elbow anatomical axis is aligned with the center of the isokinetic device rotating axis (Figure 2). The maximal range of movement of the elbow was measured by the isokinetic dynamometer from maximum flexion to maximum extension as much as possible. Each subject was required to complete 1 pre-test and 5 post-tests. Three repetitions of maximal elbow extension and flexion at a speed rate of 60°/sec were performed starting with extension during each test. Verbal encouragement was used to urge them to perform as fast as they could. After the pretest, the participants of RA group received acupuncture treatment at the acupoints for 20 min by the experienced acupuncturist, while the participants of SA group received acupuncture treatment near the acupoints for 20 min. Data were collected before the treatment (pre), immediately after the end of the treatment (post1), and repeated after 7 min (post2), 14 min (post3), 21 min (post4), and 28 min (post5). Isokinetic, dynamometric, and sEMG measurements were recorded before and after treatment by the same assessors in all participants. The experimental process is shown in Figure 3.

### 2.5. Data Analysis

In this study, kinematic data were acquired using a computer with the Con-Trex® software adopted dynamometer. Isokinetic parameters including torque, work, power, and speed were recorded with a high correlation with our study confirmed. A total of 6 s of continuous maximum isometric contraction was collected to determine MVC, and in the first second, sEMG was discarded in consideration of the delayed transition period between muscle force and signal performance. The sEMG signals were recorded as the isometric maximum voluntary contraction (IMVC) of four muscles, and the values were exhibited as IMVC percentage. A fourth-order low-pass Butterworth digital filter was applied to the sEMG data with a cutoff frequency at 50 Hz before further analysis. The stiffness of the elbow joint was calculated using the following formula:

(1) $$\begin{array}{c} K_{\text{elbow}}=\frac{\Delta M_{\text{elbow}}}{\Delta \theta_{\text{elbow}}}, \end{array}$$

where the change in the joint moment between maximum elbow Flex/Ext is defined as Δ*M*_elbow_ and the change in the joint angle between maximum elbow Flex/Ext is defined as Δ*θ*_elbow_. The joint moment was normalized to the participant's body weight.

### 2.6. Statistical Analysis

All statistical tests were conducted using MATLAB (version R2016a; MathWorks, Inc., Natick, MA). Two-way mixed ANOVA was used to evaluate the effect of group (SA and TA) and time (pre, post1, post2, post3, post4, and post5) on the isokinetic parameters, sEMG data, and joint stiffness for elbow extension/flexion muscle endurance followed by Bonferroni post hoc test to perform pairwise comparisons between time points. Cohen's d was also included to assess the effect size (ES); a difference of 0.2–0.5 is regarded as small, between 0.5 and 0.8 as medium, and greater than 0.8 as large [18]. Data were presented as mean with standard deviation (mean ± SD). The significant threshold was set at *p* < 0.05.

## 3. Results

Thirty-six female subjects completed the study with no adverse reactions and no dropouts during the procedure.

### 3.1. Kinematic Isokinetic Parameters of Elbow Flexion-Extension Muscle Groups

**Figure 4:**
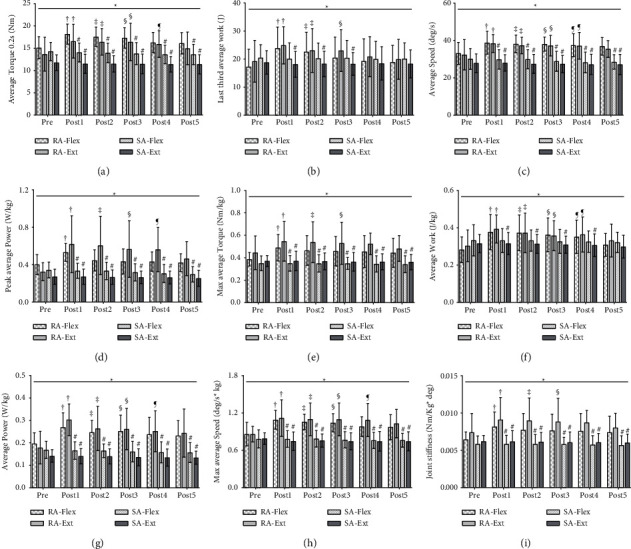
Mean (SD) of isokinetic parameters for elbow flexion-extension endurance before and after acupuncture. “*∗*”: statistically significant interaction effect of isokinetic parameters from pre to post5 at the RA/SA group (*p* < 0.05). “†” indicates a significant difference between pre and post1 (*p* < 0.05). “‡” indicates a significant difference between pre and post2 (*p* < 0.05). “§” indicates a significant difference between pre and post3 (*p* < 0.05). “¶” indicates a significant difference between pre and post4 (*p* < 0.05). “*δ*” indicates a significant difference between pre and post5 (*p* < 0.05). “#” indicates a significant difference between RA and SA at any time points (*p* < 0.05). Real acupuncture group: RA and sham acupuncture group: SA.

The results in Figure 4 indicated that there were significant interaction effects between group*∗*times on the average torque Flex/Ext (*p*=0.009, *p*=0.006), last third average work Flex/Ext (*p*=0.005, *p*=0.006), average speed Flex/Ext (*p*=0.001, *p*=0.006), peak average power Flex/Ext (per kg) (*p*=0.041, *p*=0.008), max average torque Flex/Ext (per kg) (*p*=0.043, *p*=0.045), average work Flex/Ext (per kg) (*p*=0.003, *p*=0.038), average power Flex/Ext (per kg) (*p*=0.004, *p*=0.006), max average speed Flex/Ext (per kg) (*p*=0.007, *p* < 0.001). Therefore, real acupuncture did improve elbow flexor and extensor endurance by analyzing isokinetic parameter values. Specifically, the simple main effect of the RA group was also significantly different on these parameters from pre to post5 (all *p* < 0.05).

Compared with the pre, Bonferroni post hoc tests revealed that average speed Flex (Figure 4(c)) and average work Flex/kg (Figure 4(f)) were significantly increased at post1, post2, post3, and post4 (all *p* < 0.05; ES varying from 0.84 to 0.93). Hence, the time effect lasted from post1 to post4 after acupuncture intervention. Average torque Flex 0.2 s (Figure 4(a)), average power Flex/kg (Figure 4(g)), and max average speed Flex/kg (Figure 4(h)) were significantly increased at post1, post2, and post3 (all *p* < 0.05; ES varying from 0.83 to 1.04). Hence, the time effect lasted from post1 to post3 after acupuncture intervention. The last third average work Flex (Figure 4(b)) was significantly increased at post1 and post2 (all *p* < 0.05; ES varying from 0.88 to 0.90). Hence, the time effect lasted from post1 to post2 after acupuncture intervention. Peak average power Flex/kg (Figure 4(d); *p*=0.028; ES = 0.87) and max average torque Flex/kg (Figure 4(e); *p*=0.042; ES = 0.82) were only significantly increased at post1 with short lasted time.

Compared with the pre, Bonferroni post hoc tests revealed that average torque Ext 02 s (Figure 4(a)), average speed Ext (Figure 4(c)), peak average power Ext/kg (Figure 4(d)), average work Ext/kg (Figure 4(f)), average power Ext/kg (Figure 4(g)), and max average speed Ext/kg (Figure 4(h)) were significantly increased at post1, post2, post3, and post4 (all *p* < 0.05; ES varying from 0.81 to 1.56). Hence, the time effect lasted from post1 to post4 after acupuncture intervention. Last third average work Ext (Figure 4(b)) and max average torque Ext/kg (Figure 4(e)) were significantly increased at post1, post2, and post3 (all *p* < 0.05; ES varying from 0.87 to 0.97). Hence, the time effect lasted from post1 to post3 after acupuncture intervention. However, there was no significant difference over time points in the SA group.

Compared with the SA group, average torque Flex 02 s (Figure 4(a)), average speed flex (Figure 4(c)), peak average power flex/kg (Figure 4(d)), max average torque flex/kg (Figure 4(e)), average power flex/kg (Figure 4(g)), and max average speed Flex/kg (Figure 4(h)) were significantly different at post1, post2, post3, post4, and post5 (all *p* < 0.05; ES varying from 0.96 to 2.24) in the RA group. Meanwhile, no significant difference were found in last third average work Flex (Figure 4(b)) and average work Flex/kg (Figure 4(f)) at any time points in the RA group.

Compared with the SA group, average torque Ext 02s (Figure 4(a)), average speed Ext (Figure 4(c)), peak average power Ext/kg (Figure 4(d)), max average torque Ext/kg (Figure 4(e)), average power Ext/kg (Figure 4(g)), and max average speed Ext/kg (Figure 4(h)) were significantly different at post1, post2, post3, post4, and post5 (all *p* < 0.01; ES varying from 1.14 to 2.95) in the RA group. Meanwhile, last third average work Ext (Figure 4(b)) and average work Ext/kg (Figure 4(f)) were significantly different at post1, post2, and post3/post4 (all *p* < 0.05; ES varying from 0.71 to 1.19) in the RA group.

### 3.2. sEMG

**Figure 5:**
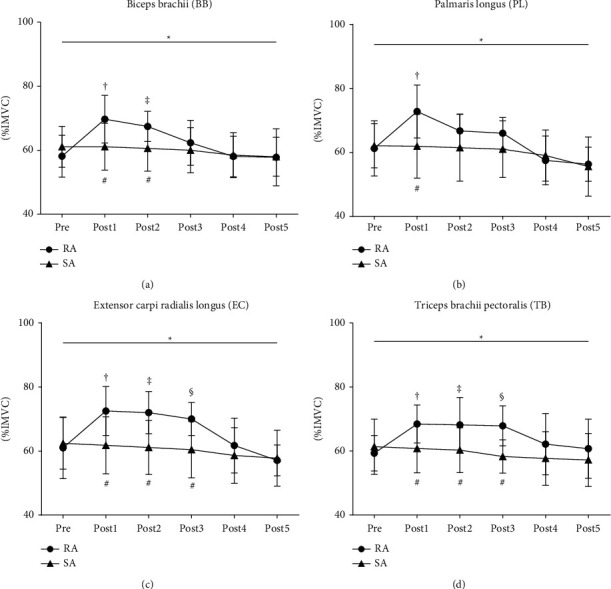
Mean (SD) of sEMG data (%MVC) for elbow flexion-extension endurance before and after acupuncture. “*∗*”: statistically significant interaction effect of isokinetic parameters from pre to post5 at RA/SA group (*p* < 0.05). “†” indicates a significant difference between pre and post1 (*p* < 0.05). “‡” indicates a significant difference between pre and post2 (*p* < 0.05). “§” indicates a significant difference between pre and post3 (*p* < 0.05). “¶” indicates a significant difference between pre and post4 (*p* < 0.05). “*δ*” indicates a significant difference between pre and post5 (*p* < 0.05). “#” indicates a significant difference between RA and SA at any time points (*p* < 0.05). Real acupuncture group: RA and sham acupuncture group: SA.

The results in Figure 5 indicated that there were significant interaction effects between group*∗*times on the sEMG values of BB (*p*=0.003), PL (*p*=0.015), EC (*p*=0.001), and TB (*p*=0.031) muscles. Therefore, real acupuncture did improve elbow flexor and extensor endurance by analyzing sEMG values. Specifically, the simple main effect of the RA group was also significantly different on the four muscles over time points (all *p* < 0.001).

Compared with the pre, Bonferroni post hoc tests revealed that BB sEMG value (Figure 5(a)) was significantly increased at post1 and post2 (all *p* < 0.01; ES varying from 0.99 to 1.02). PL sEMG value (Figure 5(b)) was significantly increased at post1 (*p*=0.015; ES = 0.93); EC sEMG value (Figure 5(c)) and TB sEMG value (Figure 5(d)) were significantly increased at post1, post2, and post3 (all *p* < 0.05; ES varying from 0.84 to 0.98). However, there was no significant difference over time points in the SA group.

Compared with the SA group, BB sEMG value (Figure 5(a)) was significantly different at post1 and post2, respectively (all *p* < 0.01; ES varying from 1.14 to 1.17); PL sEMG value (Figure 5(b)) was significantly different at post1 (*p* < 0.01; ES = 1.19); EC and TB sEMG values (Figures 5(c) and 5(d)) were significantly different at post1, post2, and post3 (all *p* < 0.01, ES varying from 1.01 to 1.65) in the RA group after acupuncture intervention.

### 3.3. Joint Stiffness

The results in Figure 4(i) indicated that there were significant interaction effects between group*∗*times on the joint stiffness Flex/Ext (*p*=0.043, *p*=0.045). Therefore, real acupuncture did improve elbow flexor and extensor endurance by analyzing elbow joint stiffness. Specifically, the simple main effect of the RA group was also significantly different on these parameters from pre to post5 (all *p* < 0.05). Compared with the pre, Bonferroni post hoc tests revealed that joint stiffness Flex was significantly increased at post1 (*p*=0.042; ES = 0.82); joint stiffness Ext was significantly increased at post1, post2, and post3 (all *p* < 0.05; ES varying from 0.94 to 0.97). Hence, the time effect lasting from post1 to post3 in joint stiffness Ext was longer than joint stiffness Flex after real acupuncture intervention. However, there was no significant difference over time points in the SA group. Compared with the SA group, joint stiffness Flex/Ext (ES varying from 0.96 to 1.90) were significantly different at post1, post2, post3, post4, and post5 (all *p* < 0.01) in the RA group.

## 4. Discussion

After RA at Quchi (LI11), Shousanli (LI10), Hegu (LI4), Xiaohai (SI8), Tianjing (SJ10), and Waiguan (SJ5) acupoints, the electromyographic values of biceps brachii, palmaris longus, extensor carpi radialis longus, and triceps brachii pectoralis of elbow extension-flexion; the joint stiffness of elbow extension-flexion; and the isokinetic parameter of elbow extension-flexion at post1 were higher than pre, indicating that RA could immediately improve the forearm muscle endurance in healthy women compared with the SA, and the effect continued to increase about 7–21 min (post2, post3, and post4) until the effect decreased or disappeared.

### 4.1. Analysis of Isokinetic Data

#### 4.1.1. Work and Power

The average work and average power of elbow extension/flexion at post1 were higher than pre after RA. Acupuncture regulates the conduction velocity of muscle fibers by stimulating nerves [19]; the muscle can recruit more motor units and increase the firing rate of activated motor units when the nerve is stimulated [20]. Therefore, acupuncture may increase the contraction and conduction velocity of muscle fibers and thereby increase the average work/power at post1 of the elbow extension-flexion muscle group in this study. In addition, acupuncture may induce the PAP effect by stimulating nerves to increase the recruitment of high-speed motoneurons; there are two common mechanisms of the PAP effect: one is the increased sensitivity of actin and myosin on Ca^2+^ release due to the phosphorylation of myosin-regulated light chains, and the other is the increased excitability of *α*-motoneurons reflected by H reflex changes [8]. Endurance athletes exhibit better PAP-fatigue balance after submaximal conditioning activities [21]; the PAP effect can counteract the peak force loss after fatigue and increase the power output and work capacity of the motor units [22]. Therefore, in this study, another reason for the increased work and power in post1 of the elbow extension-flexion muscle group may be that acupuncture stimulates the nerve to induce the PAP effect and is more conducive to balance PAP-fatigue after submaximal conditioning activity, thereby enhancing the antifatigue performance in endurance exercises.

#### 4.1.2. Torque and Speed

The average torque and average speed of elbow extension/flexion at post1 were higher than pre after RA. Motoneuron activity is regulated through the spinal and supraspinal level inputs, and the recruitment of motoneuron is the basis for the production of muscle strength [23]. Past studies have shown that increased reflex excitability in the spinal and supraspinal level may induce the generation of dorsiflexion to plantar flexion peak torque and increase the rate of torque development in isokinetic ankle movements [24]; neurophysiological evidence of H reflex indicates that acupuncture can increase the excitability of spinal motoneurons [25]. At the same time, acupuncture may increase the excitability of *α*-motor neurons by stimulating the nervous system and thereby induce the PAP effect [8, 13]. Therefore, the reason for the increased average torque and speed of elbow extension-flexion at post1 in this study may be that acupuncture increased the excitability of motoneurons at the spinal and supraspinal levels. In addition, the gradual increase of torque can increase the joint stiffness by controlling the joint rotation [26]; in this study, the increased torque of elbow extension/flexion may contribute to the increase of elbow joint stiffness and complete flexion-extension movement.

### 4.2. Analysis of sEMG Data

The electromyographic values of the biceps brachii, palmaris longus, extensor carpi radialis longus, and triceps brachii pectoralis of elbow extension/flexion at post1 were higher than pre after RA. In this study, the location of acupuncture was at the anatomical location of acupoints, where there were afferent and efferent fibers of the body that controlled the skin, connective tissue, and skeletal muscle. Acupuncture at acupoints may activate various mechanoreceptors in the superficial skin and deep muscle tissues to increase the excitability of nerve fibers [27]. Past studies have also obtained some conclusions similar to this study. Acupuncture at Quchi (LI11) acupoint can more significantly increase the sEMG value of the brachioradialis muscle in the right upper limb, and the increase of sEMG value may be beneficial to strengthen brachioradialis muscle endurance [28]; Needling motor points of extensor-flexor can increase sEMG peak value and the integrated electromyogram (IEMG) of the muscles around the wrist joint of the upper limb and thereby increase the strength of the extension-flexion muscle group [29]. Therefore, acupuncture may increase the excitability of nerve fibers in muscle tissue near a specific acupoint area and increase the EMG activity in this study.

### 4.3. Analysis of Joint Stiffness

The joint stiffness of elbow extension/flexion at post1 was higher than pre after RA. Joint stiffness is related to the co-activation of the agonist and antagonist, and the activation of the neuromuscular system may contribute to the increase of joint torque and stiffness [30, 31]. The co-contraction of agonist and antagonist muscles is often used to enhance joint mechanical stability and increase the joint stiffness. Previous studies have found that the co-contraction of wrist flexor-extensor muscles can enhance wrist joint stiffness and thereby improve wrist mechanical impedance [32]. In this study, the increase at post1 of elbow joint stiffness after acupuncture may be caused by the co-contraction of elbow flexion-extension muscles. Better contraction of muscles depends on muscle activation effectively, and acupuncture may improve the efficiency of muscle contraction by activating the excitability of nerve fibers in muscle tissue. Therefore, in this study, the recruitment of motor units increased after RA to enhance the EMG activity of the forearm muscles and may increase the joint stiffness at post1 of elbow extension/flexion.

### 4.4. The Effect of Acupuncture Time-Dependent of Muscle Endurance

To summarize up, the increase at post1 compared to the preindicated that the forearm muscle endurance can be immediately improved after RA, which is consistent with the results of previous studies that acupuncture can improve the exercise endurance of upper limb muscles by analyzing the EMG of biceps brachii [33]. The average torque, work power, and speed (per kg) of the elbow flexion muscle group increased after RA at post1, post2, and post3/post4 compared with pre, indicating that the muscle endurance of the forearm flexor continued to increase about 7–21 min after RA. Past studies have confirmed the existence of acupuncture posteffect; acupuncture removal may cause a strong stimulus-induced response; and the lasting time of post-effect is about 10 min [34]. The time-effect of acupuncture on specific points around the shoulder joint to improve the explosive force is about 10 min [35]. The response characteristics that weaken or disappeared over time by stimulating the deep structure of human acupoints are closely related to the “*De-qi*” sensation after acupuncture; the duration of the “*De-qi*” sensation may induce long-term plastic changes in the central nervous system and affect the efficacy of acupuncture [19, 36]. Therefore, in this study, the muscle endurance improvement of forearm flexor was time-dependent, which may be that acupuncture stimulated nerves to induce the “*De-qi*” response and affect the muscle function about 7–21 min; the flexion muscles of the elbow joint may continue to “*De-qi*” after acupuncture removal, making the increase of torque, work, power, and speed with time effect. Moreover, the average torque work power, and speed (per kg) of the elbow extension muscle group increased at post1, post2, and post3/post4 after RA compared with pre, indicating that the sustained enhancement time of forearm extensor endurance after RA is about 14–21 min. In addition, the PAP effect may be beneficial to enhance the duration of “*De-qi*”; previous studies have found that the neuromuscular phenomenon caused by the PAP effect can increase the speed of force development; and the maximum power and torque may be the result of enhanced myosin phosphorylation [37, 38]. The PAP effect increases immediately after the maximum voluntary contraction and disappears completely after 10–15 min [39]. However, the stimulation of different acupoints in the same spinal segment can elicit different functional magnetic resonance imaging (fMRI) activation patterns in the brain [40]; different acupuncture acupoints have different nerve innervations; and the intensity of individual “*De-qi*” sensation caused by acupuncture is also different [41]. In this study, the muscle endurance of flexor was increased about 7–21 min after RA, while the time effect of extensor muscles was increased about 14–21 min. It may be that the acupoints selected in this study were mainly distributed in the forearm extension muscle group and caused a greater extent activation of the elbow extension muscle group by acupuncture, inducing the PAP effect and thereby prolonging the “*De-qi*” time of acupuncture.

### 4.5. Limitations

This study has certain limitations. The use of an isokinetic dynamometer to record changes in isokinetic parameters does not analyze the actual movements of elbow joint flexion-extension, and the isokinetic flexion-extension test is different from the movement of the actual competition field due to the movement restriction. Future research on acupuncture may be used actual movements as a test to clarify the effect on sports performance. In addition, the SA group also had some limitations, even though sham acupuncture was still identified as the primary method for determining acupuncture treatment [42]. The sham acupuncture may also cause nerve pain that induces muscle-stimulating effects.

## 5. Conclusion

In this study, the results showed that RA can improve the endurance performance of upper limb muscles immediately compared with the SA and has a time effect by activating the neuromuscular system. After RA for 20 min, the sEMG values of biceps brachii, palmaris longus, extensor carpi radialis longus, and triceps brachii pectoralis of elbow extension-flexion increased; the joint stiffness of elbow extension-flexion increased; and the isokinetic parameters of the average torque, work, power, and speed in elbow extension muscles were increased about 14–21 min, while in flexion muscles were increased about 7–21 min by comparing differences between post1, post2, post3, post4, post5, and pre. In addition, the longer time-dependent characteristics of the elbow extensors compared with the flexors may be related to the specificity of the selected acupuncture acupoints. This study provides a theoretical basis for clinical and sports medicine research on acupuncture, which can be used as an alternative therapy to improve sports performance in upper limb endurance exercises, in the future; this research method can be considered to improve the performance of antifatigue and enhance the forearm muscle endurance.

## Data Availability

The data used to support the findings of this study are available from the corresponding authors upon request.
